# Insomnia during pregnancy: Diagnosis and Rational Interventions

**DOI:** 10.12669/pjms.324.10421

**Published:** 2016

**Authors:** Ali M. Hashmi, Shashi K. Bhatia, Subhash K. Bhatia, Imran S. Khawaja

**Affiliations:** 1 Ali M. Hashmi, MBBS, MD. Diplomate American Board of Psychiatry and Neurology, Associate Professor of Psychiatry, King Edward Medical University/Mayo Hospital, Lahore, Pakistan; 2 Shashi K. Bhatia, MD; FACPSYCH; FAACAP Diplomate, American Board of Psychiatry and Neurology in General and Child and Adolescent Psychiatry, Professor and Division Director, Department of Child and Adolescent Psychiatry, Professor, Department of Pediatrics, Creighton University School of Medicine, Omaha, Nebraska, USA; 3 Subhash K. Bhatia, MD; FACPSYCH, MAMS. Diplomate, American Board Psychiatry and Neurology in General Psychiatry and in the Subspecialties of Addiction, Forensic and Geriatric Psychiatry, Distinguished Professor and Senior Associate Chair for Academic Affairs and Faculty Development, Creighton University Department of Psychiatry Adjunct Professor, University of Nebraska College of Medicine, Omaha, NE 68105, USA; 4 Imran S. Khawaja MD, FAASM. Medical Director, Center for Sleep Medicine, VA Medical Center, Dallas, Texas, Associate Professor of Psychiatry and Neurology, UT Southwestern School of Medicine, USA

**Keywords:** Insomnia, Obstructive Sleep apnea, Pregnancy

## Abstract

Sleep disturbances are common in pregnancy. Insomnia is a frequent sleep disturbance experienced by pregnant women which can be primary or due to co-morbid conditions. The differential diagnosis of insomnia in pregnancy includes anxiety disorders, mood disorders, breathing related sleep disorders and restless legs syndrome. Early interventions to treat the sleep disturbance are recommended to avoid adverse pregnancy outcomes. Management strategies include improving sleep hygiene, behavioral therapies, and pharmacotherapy. The risks of pharmacotherapy must be weighed against their benefits due to the possible risk of teratogenicity associated with some medications.

**Methods:**

We searched PubMed and Google Scholar employing a combination of key words: pregnancy, sleep disturbances, Obstructive Sleep Apnea, Sleep disorders and insomnia. We included original studies, review articles, meta-analysis and systematic reviews in our search prioritizing articles from the last 10-15 years. Articles older than 15 years were only included if their findings had not been superseded by more recent data. Further selection of articles was done from bibliographies and references of selected articles.

**Conclusion:**

Sleep disturbances in pregnancy are common and cause considerable morbidity. Management includes a combination of non-pharmacological and pharmacological treatments carefully weighing the risks and benefits of each for the expectant mother and fetus.

## INTRODUCTION

Sleep disturbances are common in pregnancy. A US National Sleep Foundation’s Women and Sleep Survey in 1998 found 78% of women reported disturbed sleep during pregnancy and 15% of women developed Restless Legs Syndrome (RLS) during 3^rd^ trimester of pregnancy. Additionally, 15% of pregnant or recently pregnant women reported one weekday nap and 60% women reported at least one weekend nap.

Sleep duration and quality related changes in pregnancy may be due to many proposed and interrelated mechanism like hormonal, physiologic, metabolic, psychological and posture related changes.

For example during first trimester, a rise in progesterone levels may cause excessive day time sleepiness, decreased muscle tone, increased risk of sleep apnea, snoring and sleep interruptions. Frequent trips to the bathroom, nausea and vomiting, pregnancy related discomfort like back pain, fetal movements and gastro-esophageal reflux can also impair the quality of sleep.

Anxiety during pregnancy may be further amplified by concerns about labor, delivery and its outcome. Poor quality sleep is not only a core feature of prenatal, intra-natal and postpartum depression, but also a risk factor for mood disturbances in pregnancy. After child birth it becomes even harder for new mothers to have a good night’s sleep.

Sleep disturbances affect health and quality of life and may also negatively influence obstetric outcomes. A recent study at University of California, San Francisco found that women who slept less than 6 hours per night were more likely to have longer labor and were 4.5 times more likely to have a cesarean section.

Both non-pharmacological and pharmacological interventions may alleviate sleep disturbances. This review is intended to provide practitioners with an understanding of sleep changes in pregnancy and guide them in rational approaches to their management.

### Sleep Alterations during Pregnancy

Sleep related problems are common during pregnancy including insomnia, RLS, sleep apnea, nighttime gastro-esophageal reflux disease (GERD), back pain, quickening and frequent nighttime urination.

### First Trimester

Sleep problems and changes in sleep patterns start during the first trimester of pregnancy^1^ most likely influenced by the rapid changes in reproductive hormone levels. Levels of progesterone rise throughout pregnancy. At 36 weeks progesterone levels are 10 times greater than peak menstrual cycle levels. Women during first trimester take day time naps in part due to fatigue.

In animal studies, progesterone administration has been observed to have sedating effects, to reduce wakefulness, shorten the latency and increase the duration of non-rapid eye movement (NREM) sleep. Estrogen reduces the amount of Rapid Eye Movement (REM) sleep.^2^ Progesterone metabolites impact brain gamma amino butyric acid-A(GABA-A) receptors^2^ which are thought to drive these sleep changes.

In animal studies, estrogen selectively suppresses REM sleep^3^ an effect possibly due to increased brainstem nor-epinephrine turnover.^4^ However, increased REM sleep has been observed in human studies of peri-menopausal women receiving estrogen replacement therapy,^5^ making it difficult to understand the specific effects of estrogen on sleep during human pregnancy. In an animal model, total sleep time increases during pregnancy, with an early but transient increase in REM duration, a sustained increase in NREM sleep over the course of pregnancy, and increased diurnal sleep during late gestation.^6^

In human pregnancy, hypersomnolence is a common complaint during the first trimester.^7^ Corresponding to this period of increased sleepiness, women surveyed about their sleep habits during pregnancy reported an average increase of 0.7 hours of sleep duration during the first trimester, compared to the pre-pregnancy period.^8^

Similarly, a mean increase of more than 30 minutes of nighttime sleep was noted at 11 to 12 weeks of gestation in 33 women who underwent in-home polysomnography prior to conception and during each trimester of pregnancy.^1^ During first trimester Stage 1 of NREM sleep increases whereas stage 3 of NREM decreases and sleep efficiency decreases compared to the pre-pregnancy period.^1^ Sleep during first trimester is also disturbed due to fatigue as well as nausea or vomiting.^1^

### Second and Third Trimester

By late in the second trimester (23-24 weeks of gestation), total night-time sleep time falls.^1^ There is an increased amount of stage 3 NREM sleep compared to the first trimester with a corresponding increase in complaints of interrupted sleep due to nocturnal GERD.^9^

During the third trimester, the majority of women have sleep difficulties. Less than 2% report no nocturnal awakenings.^8^ There is reduction in the percentage of REM and Stage 3 NREM sleep and an increase in stage 1 NREM sleep.^10^ Despite increased wake time after sleep onset and reduced nighttime sleep time compared to the first 2 trimesters, total sleep time normalizes or increases to approximately pre-pregnancy sleep level. There is no evidence of a shift in circadian phase (e.g. delayed sleep phase or advanced sleep phase) with melatonin levels showing a diurnal rhythm.

A majority of women experience sleep problems in 3^rd^ trimester with over 98% reporting nocturnal awakenings. There is a rise in Stage 1 NREM and reduction in Stage 3 and REM parts of sleep. Despite these changes and reduced sleep time as compared to first 2 trimesters, total sleep time normalizes to almost pre-pregnancy levels. There is no evidence of changes in circadian rhythm with melatonin levels showing a diurnal rhythm. In 3^rd^ trimester, sleep disturbances are due to general discomfort caused by backache, urinary frequency, fetal movements, GERD and leg discomfort.

### Postpartum period

Sleep problems increase in the first 6 months after child birth with total nocturnal sleep time of less than 6 hours.^11^ Sleep efficiency improves over time as the infant’s circadian rhythm matures. Women who breastfeed have more stage N3 sleep (third phase of NREM sleep) than those who do not, which could be attributed to prolactin’s effect on stage N3 sleep.^12^ There is shortened latency to stage REM sleep at 1 month postpartum, which could be attributed to progesterone returning to pre-pregnancy level or sleep loss in the postpartum period.^13^

### Insomnia in pregnancy

Insomnia is defined both as a symptom and as a disorder. As a symptom this clinical condition is quite common in practice, however, as a diagnosis, insomnia has multiple defined sub-classifications in DSM 5 (Diagnostic and Statistical Manual-5th Edition) (Table-I).^14^

**Table-I:** Subclassification of sleep disturbances according to DSM-5.

Type of Sleep Disorder	Diagnostic Criteria	Risk Factors
Breathing Related Sleep Disorders	In DSM-5, the breathing related sleep disorders are classified into three relatively distinct disorders: obstructive sleep apnea hypopnea, central sleep apnea and sleep related hypoventilation.	
Obstructive Sleep Apnea Hypopnea	Polysomnography (PSG) evidence of at least five obstructive apneas or hypopneas per hour of sleep and either of the following symptoms: 1. (a) Nocturnal breathing disturbances: Snoring, snorting/gasping, breathing pauses during sleep. (b) Daytime sleepiness, fatigue, or unrefreshing sleep despite sufficient opportunities to sleep that is not better explained by another mental disorder (including a sleep disorder) and is not attributable to another medical condition. 2. Evidence by polysomnography of 15 or more obstructive apneas and/or hypopneas per hour of sleep regardless of accompanying symptoms.	Obesity Excessive gestational weight gain Smoking Drug or alcohol use Prior history of snoring Large neck circumference
Restless Legs Syndrome	A. An urge to move the legs accompanied by or in response to uncomfortable or unpleasant sensation in legs characterized by: 1) The urge to move the legs begins or worsens during periods of inactivity or rest. 2) The urge to move the legs is partially or fully relieved by movement. 3) The urge to move the legs is worse in evening and night than at day, or occurs only in the evening or at night. B. The symptoms occurs >3 times/week or have persisted for at least 3 months C. Accompanied by significant impairment or distress in social, behavioral, educational, academic and other areas of functioning. D. The symptoms in A are not attributed to any medical disorder (e.g., arthritis, leg cramps, leg ischemia) or any other mental disorder (e.g. habitual foot tapping) E. Symptoms are not attributable to the physical effects of drugs of abuse or medication.	RLS prior to pregnancy Family history of RLS Folate/ferritin deficiency Childhood history of growing pains Obesity
Insomnia (Primary or comorbid) Insomnia (primary or comorbid	A predominant complaint of dissatisfaction with sleep quantity or quality, associated with one (or more) of the following symptoms: 1. Difficulty initiating sleep. (In children, this may manifest as difficulty initiating sleep without caregiver intervention). 2. Difficulty maintaining sleep, characterized by frequent awakenings or problems returning to sleep after awakenings. (In children, this may manifest as difficulty returning to sleep without caregiver intervention.) 3. Early-morning awakening with inability to return to sleep. B. The sleep disturbance causes clinically significant distress or impairment in social, occupational, educational, academic, behavioral, or other important areas of functioning.	Chronic Pain Passive smoke Exposure Neurological disorder African American Shift Worker Depression
Insomnia (Primary or comorbid	C. The sleep difficulty occurs at least 3 nights per week. D. The sleep difficulty is present for at least 3 months. E. The sleep difficulty occurs despite adequate opportunity for sleep. F. The insomnia is not better explained by and does not occur exclusively during the course of another sleep-wake disorder (e.g., narcolepsy, a breathing-related sleep disorder, a circadian rhythm sleep-wake disorder, a parasomnia). G. The insomnia is not attributable to the physiological effects of a substance (e.g., a drug of abuse, a medication). H. Coexisting mental disorders and medical conditions do not adequately explain the predominant complaint of insomnia.	Stress

A majority of women experience insomnia during pregnancy with rates as high as 80%.^15^ Insomnia is worse in the third trimester.^15^ A diagnosis is usually made by clinical history which includes screening for common sleep disorders seen in pregnant women.

Because of the common co-morbid nature of insomnia, the DSM-5 recommends using the term “Insomnia disorder” instead of secondary insomnia since the term “secondary” suggests that treating the primary disorder or problem is all that’s needed to treat insomnia. Common pregnancy related complaints like back pain, nocturia, fetal movement, breast tenderness and leg cramps can negatively affect sleep quality and quantity.^15^ However, treating these problems does not necessarily mean that insomnia will automatically get better.

Daytime effects of insomnia include hypersomnia, fatigue and mood changes. Insomnia can also negatively impact partner relationship and interfere with mother-infant bonding. Moreover, sleep disturbances in 3^rd^ trimester are associated with increased perception of labor pain, longer labor and increased operative births. Patients with insomnia have high pro-inflammatory cytokines which is also seen in postpartum depression, preterm birth and other pregnancy complications. Clinicians should address the sleep disturbances promptly because it puts the pregnant women at higher risk of complications like depression in late third trimester or after child birth.

### Differential diagnosis of insomnia in pregnancy

### Anxiety disorders

Sleep disturbance assessment should include careful screening of primary mood disorders like major depressive disorder (MDD) or bipolar disorder or primary anxiety disorders like generalized anxiety disorder (GAD), post-traumatic stress disorder (PTSD), panic disorder or obsessive compulsive disorder (OCD) because these conditions can present as prenatal insomnia.

As with MDD, diagnostic criteria for conditions such as GAD may overlap with common symptoms of pregnancy including being easily fatigued, difficulty concentrating, emotional reactivity, and muscle tension. For the diagnosis of GAD, patients also manifest excessive anxiety and worry that is difficult to control for at least 6 months and cause clinically significant distress and impairment. Similarly, difficulty falling or staying asleep and restless sleep are included in the diagnostic criteria for PTSD but patients with PTSD also have other symptoms such as hyperarousal, increased reactivity, flashbacks, and avoidance of traumatic stimuli.

### Mood disorders

According to DSM-5, sleep disturbances are an important feature of MDD. They are also central features of manic and hypomanic episodes which are an important consideration in evaluating pregnant women with sleep problems. Due to the overlap of symptoms of pregnancy with neurovegetative symptoms of depression, the diagnosis of mood disorders becomes very challenging.

Clinicians can rely on several elements to diagnose primary mood disorder. For instance, pregnant women with depression experience anhedonia (diminished interest or pleasure in all or almost all activities) nearly every day. Other symptoms like psychomotor retardation, feeling of worthlessness, excessive or inappropriate guilt and recurrent thoughts of suicide are primarily experienced by pregnant women with depression. Moreover, assessment of time course, frequency and severity of symptoms can differentiate between pregnancy symptoms and symptoms of MDD.

According to DSM-5, symptoms should not only occur for most days over a 2 week period but should also cause significant distress in social, occupational and other areas of functioning. Before considering a diagnosis of MDD, patient should be screened for a past history of hypomania or mania (days/weeks of expansive thought, decreased need for sleep, impulsivity, racing thoughts, talkativeness etc.). Pregnant women can present with MDD, but a past history of mania or hypomania would suggest Bipolar Disorder, leading to different pharmacological treatments like mood stabilizers before the postpartum period, a time with higher risk of recurrent mood episodes.

### Sleep Disorders

a) ***Obstructive Sleep Apnea-Hypopnea (OSAH)*** Obstructive sleep apnea is a breathing related sleep disorder characterized by repeated episodes of apnea (cessation of breathing) or hypopnea (decrease in the flow of breathing accompanied by oxygen desaturation) secondary to obstruction of airflow in the upper airway.^7^,^14^,^20^ The association between breathing related sleep disorders and hypertension, cardiovascular disease, diabetes and chronic pain related conditions like fibromyalgia is well established in the general adult population.^20^ There is a paucity of data about these conditions in pregnant women. The estimated prevalence of OSAH is 2% in non-pregnant women.^15^ In contrast, the prevalence of OSAH in pregnant women has been reported to be higher and is reported to be between 10-25%.^15^ Normal physiological and hormonal changes in pregnancy (including weight gain, edema and diaphragmatic displacement secondary to enlarging uterus) can contribute to breathing related sleep difficulties. In addition, higher circulating levels of estrogen cause edema of mucous membranes which can lead to nasal congestion and pharyngeal constriction, another cause of breathing related sleep disorders.
b) ***Restless leg syndrome (RLS)*** RLS is another condition that can contribute to insomnia in pregnancy (Table-I).^21^ Restless legs syndrome is common in the general population with an incidence of 3.4% to 10%.^21^,^22^ The incidence is much higher in pregnant women (27%-30%) with the symptoms being worse in the third trimester.^23^ For those who develop the condition during pregnancy, symptoms usually remit after delivery. In patients who suffer from RLS before pregnancy, symptoms can worsen during gestation. RLS is often under-diagnosed owing to lack of awareness of clinicians and because symptoms can be similar to leg cramps which is quite common in pregnancy.
c) A thorough sleep history which includes the diagnostic criteria of RLS can help with accurate diagnosis. Iron deficiency (ferritin) can be associated with RLS in pregnant women via changes in dopamine transporter functioning.

Untreated RLS increases risk of depressed mood, and RLS related sleep deprivation is linked to adverse effects like prolonged labor, heightened pain perception and discomfort during labor, higher rates of C-section, preterm labor and elevated inflammatory cytokines.^24^

### Management of insomnia during pregnancy

It is important that clinician should inquire about difficulties in sleep initiation, maintenance or early morning awakening and understand environmental and behavioral factors. Obtaining a complete medical history including risk factors is pivotal for diagnosis and treatment and early intervention is recommended.

### Non-pharmacologic Interventions

1. ***Sleep Hygiene and Sleep Education*** Non pharmacological interventions like sleep hygiene and education should be considered as first line strategies. (Table-II) It is heartening to know that most of the sleep problems experienced by pregnant women tend to improve with child birth. Sleep hygiene strategies can significantly improve the quality of sleep without the need to resort to medications. These include:

**Table-II:** Non-Pharmacological Interventions.

Non-Pharmacological Interventions	Comments	Evidence for Safety
Sleep Hygiene and Education	Low risk and effective	++++
Behavioral therapies	Low risk and effective	++++
  - Use dim nightlights in bathroom as bright light can make it difficult to go back to sleep
  - Drink plenty of fluids in daytime but limit their intake after 5pm to decrease frequent awakenings for urination.
  - Avoid spicy, heavy and fried foods to decrease heart burn. Over the counter antacids may be used.
  - Prefer daytime naps in the earlier part of the day, if needed.
  - If medically appropriate exercise 30 minutes every day preferably 4 to 6 hours prior to bed time.
  - If unable to sleep don’t force yourself to go to sleep. Instead get out of bed, take a warm bath and do something non-stimulating such as knitting, reading a book, etc.
  - The environment of your bedroom should be comfortable.
  - Avoid activities like eating, watching TV, playing videogames or other electronics or lengthy cell phone calls while in bed.
  - Sleep lying on the left side with knees and hips bent and pillows between the knees, under abdomen and behind the back to reduce pressure on lower back. In addition a heating pad on the back may reduce pain and improve sleep
  - For Restless Legs syndrome have your physician evaluate you for folic acid or iron deficiency.
  - Snoring is common during pregnancy. Physician intervention may be needed if there are pauses in breathing between snoring episodes indicating possible OSAH.
  - Avoid stimulants like caffeine and heavy nicotine use close to bed time
  - To reset your internal clock, go to sleep and wake up at the same time every day.
2. ***Behavioral Therapy*** Behavioral therapies for insomnia in addition to sleep hygiene and stimulus control include: relaxation, sleep restriction, cognitive therapy and cognitive behavioral therapy for insomnia (CBT-I).^25^
  - Stimulus control includes using your bed only for sleep. If unable to sleep get up and do something minimally stimulating. Staying in bed and worrying about not sleeping perpetuates insomnia.
  - Relaxation techniques like progressive muscle relaxation (PMR) which includes alternately tightening and relaxing different muscle can be used before each sleep period. Abdominal deep breathing with relaxing thoughts can also be helpful if used before each sleep period.
  - Sleep Restriction (not lying in bed for extended periods of time) prevents circadian clock shifts and is helpful in preventing insomnia. Restricting the time in bed to the amount of sleep reported on a sleep log improves sleep efficiency. Sleep efficiency is computed as time asleep divided by time in bed. Once it reaches 85 % you may increase time in bed by 15 to 30 minutes.
  - Cognitive Therapy is directed at anxiety, catastrophic thinking related to not sleeping and having the patient develop realistic expectations about duration of sleep. Some patients have predetermined ideas about the amount of sleep needed to function well. That has to be addressed through research data and patients’ past history of duration of sleep needed to function well.
  - Cognitive behavioral therapy for insomnia (CBT-I), includes daily sleep logs, a session on sleep education, two sessions with focus on stimulus control and sleep restriction followed by 2 sessions on cognitive therapy as outlined above followed by a session on sleep hygiene and a final session to integrate information from all of above session. CBT-I also includes sleep hygiene and helping patient deal with maladaptive thoughts, beliefs and behaviors like watching TV in bed. In addition to cognitive therapy, other components of CBT-I include stimulus control therapy and sleep restriction. Stimulus control helps patients establish a regular sleep/wake schedule, establishing bed and bedroom as cues for sleep and reducing association with activities that might be stimulating. Patients are asked to go to bed only when sleepy and to use their bed for sleep only. However, it is not known if these techniques improve insomnia in pregnant women as well.

### Pharmacological Interventions (Table-III)

**Table-III:** Pharmacological Interventions with Safety Profile.

**Non-Benzodiazepines**				
Drug	Pregnancy Risk	Lactation Risk	Comments	Evidence for Safety
Chloral Hydrate	C	L3	Limited Risk	+++
Eszopiclone	C	NA	Limited Risk	+++
Zaleplon	C	L2	Limited Risk	+++
Zolpidem	B	L3	Limited Risk	+++
Diphenhydramine	B	NA	Limited Risk	+++

**Benzodiazepines**				
Drug	Pregnancy Risk	Lactation Risk	Comments	Evidence/Safety

Estazolam	X	L3	Significant risk	+/-
Flurazepam	X	L3	Significant risk	+/-
Temazepam	X	L3	Significant risk	+/-
Triazolam	X	L3	Significant risk	+/-

**Tricyclic Antidepressants**				
Drug	Pregnancy Risk	Lactation Risk	Comments	Evidence/Safety

Amitriptyline	C	L2	Useif primary disorder is depression	+/-
Clomipramine	C	L2	Use if primary disorder is depression	+/-
Doxepin	C	L5	Useif primary disorder is depression	+/-
Imipramine	C	L2	Useif primary disorder is depression	+/-

**Selective Serotonin Reuptake Inhibitors**				
Drug	Pregnancy Risk	Lactation Risk	Comments	Evidence/Safety

Fluvoxamine	C	L2	Useif primary disorder is depression	+/-
Paroxetine	D	L2	Increased risk of congenital cardiac problems. Not recommended	-negative

**Other Antidepressants**				
Drug	Pregnancy Risk	Lactation Risk	Comments	Evidence/Safety

Mirtazapine	C	L2	Use if primary disorder is depression	+/-
Trazodone	C	L2	Adjunctiveuse for sleep induction	+/-
**Antipsychotics**				
Chlorpromazine	C	L3	Useif primary Disorder is psychosis	+/-
Olanzapine	C	L2	Useif primary Disorder is psychosis	+/-
Quetiapine	C		Use if primary Disorder is psychosis	+/-
**Mood Stabilizers**				
Carbamazepine	D	L2	Use only if primary diagnosis is bipolar disorder. Avoid in first trimester to prevent neural tube defects	+/-
Lamotrigine	C	L3	Use only if primary diagnosis is bipolar disorder	+/-
Lithium	D	L4	Use only if primary Diagnosis is bipolar disorder. Avoid in first trimester to prevent Epstein cardiac anomaly	+/-
Valproic Acid	D	L2	Use only if primary diagnosis is bipolar. Avoid in first trimester to prevent neural tube defects	+/-

**FDA Pregnancy Drug Safety Rating:** A=No Risk noted in controlled studies; B=Evidence of Risk in Humans; C=Risk cannot be ruled out; D= Positive Evidence of Risk; X=Contraindicated in Pregnancy. **Lactation Risk Categories:** L1=Safest; L2=Safer; L3=Moderately Safe; L4=Possibly Hazardous; L5+ Contraindicated NA= Not available. Adapted from: Armstrong C. Practice Guidelines: ACOG Guidelines on Psychiatric Medication Use During Pregnancy and Lactation. Am Fam Physician 2008; 78:772-778. ++++ Recommended, +++ May be recommended, ++Recommended only if benefits clearly out way risk,+Use with extreme caution due to risk to developing fetus, +/- Not recommended unless underlying condition is severe enough to pose significant risk to fetus and/or mother e.g. florid mania or psychosis, severe agitation, active suicidal or homicidal ideation in context of a psychiatric illness.

- Most medications for RLS pose risks to the developing fetus. Low folate and iron levels increases risk of developing RLS during pregnancy, so prenatal supplementation of vitamins and iron should be considered. Folate is better absorbed in foods like grains, cereals and bread than in pill supplements. Coffee consumption decreases, while vitamin C increases absorption of folate from food.
- Overweight or obese women, who become pregnant, gain more weight and women who report snoring should be evaluated for sleep apnea. Continuous positive airway pressure (CPAP) is a safe and effective treatment for sleep apnea during pregnancy.
- GERD can be treated with over-the-counter antacids.
- There is no over-the-counter remedy for pregnant women who experience frequent nighttime urination other than using the strategy recommended earlier.
- For moderate to severe sleep disturbance related to an underlying mood or anxiety disorder during pregnancy, pharmacological treatment is often essential to improve sleep quality and reduce risk of untreated prenatal psychiatric illness including diminished self-care, self-harm, suicide, potential impact on delivery and labor and higher risk for postpartum exacerbation.^26^ The risks should be considered during treatment discussions because providers often focus more on teratogenic risk of medications rather than risk of untreated psychiatric illness.^27^

### Hypnotics

If non-medical interventions have failed for moderate insomnia during pregnancy antihistamines like doxylamine, which is safe in pregnancy, can be used.^28^ For pregnant women with more severe insomnia, treatment with a sedating antidepressant or sedative-hypnotic may be necessary. Commonly used sedative-hypnotics like Zolpidem have limited reproductive safety data which limits their use in pregnancy.^29^-^31^

### Benzodiazepines

For severe anxiety and insomnia benzodiazepines like lorazepam can be considered. Although earlier studies have shown increased incidence of cleft lip with their usage during pregnancy, recent studies have not found any association.^32^,^33^

A recent meta-analysis^32^ showed that the risk of major congenital abnormalities was similar in children born to mothers with anxiety and depression but without any drug exposure in the first trimester when compared to children born to mothers who received diazepam, temazepam, eszopiclone or other anxiolytic/hypnotics in the first trimester indicating that prescription of these drugs during early pregnancy may be safe in terms of risk of major congenital anomalies but further studies are needed to confirm safety. Thus, informed consent from both the mother and father as to risks/benefits of these drugs is warranted. Case reports of possible withdrawal or toxicity symptoms in newborns exposed in utero to benzodiazepines have included descriptions of increased sedation, abnormal muscle tone, respiratory or sleep problems.^27^,^34^,^35^ For pregnant women struggling with extreme sleep, mood or anxiety symptoms, the benefits of using low dose benzodiazepines may outweigh these reported concerns, however, the lowest effective dose should be prescribed to lower risk of withdrawal and toxicity in infants postpartum.^27^ In summary, benzodiazepines in pregnancy should not be prescribed carelessly and without a full analysis of the risks/benefits and thorough discussion of these with both parents.

### Antidepressants

If the sleep issue in pregnancy is due to depression or anxiety disorder, antidepressants with non-pharmacologic therapy can be helpful. Sedating tricyclic antidepressants maybe a better choice because of lack of evidence of increased risk for major congenital malformations. Though concerns exists regarding the teratogenic effects of antidepressants,^36^ there is very strong evidence that antidepressants do not raise risk for congenital malformations^37^-^39^ with the possible exception of paroxetine which has been associated with cardiac defects in some^40^ but not other^41^ studies. Studies have also shown that fetal exposure to maternal depression with or without exposure to an antidepressant also has negative effects on infant health^42^ Perinatal toxicity effects like jitteriness, respiratory and feeding difficulties, and sedation have been described in cases of exposure to antidepressants but they are generally thought to be short-lived and not life threatening.^36^,^43^

### Other Pharmacological agents

Pregnant women with bipolar disorder presenting with depressive, hypomanic or manic symptoms may require a mood stabilizer along with a sedating atypical antipsychotic or benzodiazepine to regulate sleep. The risks for an evolving bipolar mood episode in pregnancy and postpartum can outweigh the known risks of certain mood stabilizers such as lamotrigine or antipsychotics, particularly older high potency neuroleptics.^44^ It goes without saying that such cases require a multidisciplinary approach and all such patients must be followed closely by both their Ob/Gyn as well as their psychiatric physicians.

## CONCLUSION

A majority of women experience sleep disturbances during pregnancy. Changes in sleep architecture result from high circulating hormone levels and physical changes associated with pregnancy. Insomnia is common during pregnancy and should be addressed early in pregnancy. Non pharmacological approaches including CBT-I are effective in treating insomnia, though studies are lacking in pregnant women. Pharmacological approaches should be considered after carefully reviewing risks and benefits of treatment versus no treatment.
